# Evaluate of Wheat Gluten as a Protein Alternative for Fish Meal and Soy Protein Concentrate in Red Spotted Grouper *Epinephelus akaara*

**DOI:** 10.3390/metabo13070832

**Published:** 2023-07-10

**Authors:** Yanbo Cheng, Yongchao Wang, Zhiyong Dong, Trond Storebakken, Guohuan Xu, Bo Shi, Yuexing Zhang

**Affiliations:** 1 National Engineering Research Center for Marine Facilities Aquaculture, Marine Science and Technology College, Zhejiang Ocean University, Zhoushan 316022, China; chengyanbo@gdim.cn (Y.C.); waych24678@163.com (Y.W.); zhiyong0310@126.com (Z.D.); 2 Department of Animal and Aquacultural Sciences, Faculty of Biosciences, Norwegian University of Life Sciences, NO-1432 Ås, Norway; trond.storebakken@nmbu.no; 3 Guangdong Provincial Key Laboratory of Microbial Culture Collection and Application, State Key Laboratory of Applied Microbiology Southern China, Institute of Microbiology, Guangdong Academy of Sciences, Guangzhou 510070, China; xuguohuan@gdim.cn

**Keywords:** *Epinephelus akaara*, fish meal, wheat gluten, soy protein concentrate

## Abstract

The aim of this study was to evaluate the effects of wheat gluten as a substitute for fish meal (FM) and soy protein concentrate (SPC) in the low-fishmeal-based extruded diet in red spotted grouper *Epinephelus akaara*. Eight isonitrogenous (441–456 g kg^−1^) and isocaloric (21.5–22.0 MJ kg^−1^) diets were produced, including the control diet (R0), three diets with 33.3, 66.7, and 100% FM being replaced by a mixture of wheat gluten, wheat, and taurine (GWT) (RF1, RF2, RF3), three diets with 33.3, 66.7, and 100% SPC replaced by GWT (RS2, RS2, RS3) and one diet with 50% FM and 50% SPC replaced by GWT (RFS). Results showed that feed intake (FI), weight gain (WG), protein retention efficiency, and liver superoxide dismutase activity increased linearly, while feed conversion ratio (FCR) decreased linearly with the decrease of dietary FM. Additionally, FI, WG, and FCR significantly increased with decreasing dietary SPC. Overall, 100% FM or 61.2% SPC can be safely replaced by wheat gluten in the red-spotted grouper diet containing 20.0% FM and 21.4% SPC.

## 1. Introduction

With the development of the aquaculture industry, aquaculture by-products such as fish waste, shrimp, molluscs, crustaceans, and sand silkworms are no longer sustainable for the development of the grouper culture industry. Therefore, high-quality formula feeds are urgently needed. Fish meal (FM) is the main protein source in aquatic feed, especially for carnivorous fish. However, the limited supply of FM and increasing demand make it necessary to investigate the substitution of FM with plant-based proteins that are highly nutritious, widely available, inexpensive, and easily stored [1]. Among all plant proteins, soy protein concentrate (SPC) has been widely used due to its nutritional characteristics (high protein and balanced amino acid profile) and relatively low content of anti-nutritional factors compared with soybean or other soybean derivatives [2]. Previous research indicated that SPC had become a widely recognised FM alternative in marine fish diets, such as rainbow trout *Oncorhynchus mykiss* [3], Japanese flounder *Paralichthys olivaceus* [4], red sea bream *Pagrus major* [5,6,7], and hybrid grouper *Epinephelus fuscoguttatus* 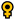 × *E. lanceolatus* 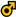 [8,9,10]. However, the price of SPC often fluctuates owing to the unstable price of soybeans; thus, finding other plant protein sources is still necessary.

Vital wheat gluten is another promising plant protein source in aquafeeds due to its high protein content and digestion, balanced amino acid composition, low content of non-starch polysaccharides, and anti-nutritional factors. Moreover, a small amount of starch can be used to accelerate ripening and as a binder during processing. Research showed that wheat gluten has similar protein digestibility to FM and SPC [11]. Currently, wheat gluten has been used as an FM substitute in rainbow trout [12,13], Atlantic halibut *Hippoglossus hippoglossus* [14], European sea bass *Dicentrarchus labrax* L. [15], large yellow croaker *Larimichthys crocea* [16], and did not cause adverse effects on growth or health.

Red spotted grouper *Epinephelus akaara*, known as red grouper or Hong Kong grouper, is commonly cultured in Southeast Asia and China. The optimum dietary protein, carbohydrate, and lipid levels for red spotted grouper were 508.3 g kg^−1^ (initial body weight = 7.88 ± 0.04 g), 76.4 g kg^−1^ (IBW = 7.79 ± 0.01 g), and less than 91.1 g kg^−1^ (IBW = 2.51 g), respectively [17,18,19]. Moreover, the optimal dietary protein requirements for orange-spotted grouper *Epinephelus coioides* were 480 (IBW = 10.7 ± 0.2 g), 521.84 (IBW = 10.02 ± 0.22 g) and 466.65 (IBW = 102.8 ± 1.02 g) g kg^−1^ reported by Luo et al. (2004), Yan et al. (2021) and Yan et al. (2020), respectively [20,21,22]. Groupers need a high protein level (about 500 g kg^−1^) for optimum growth. However, due to protein is the most expensive ingredient in the diet, some other protein sources have been used to replace fishmeal in orange-spotted grouper and pearl gentian grouper feeds, such as soybean meal, animal by-product meals, cottonseed protein concentrate, black soldier fly, soy protein concentrate, Tenebrio molitor meal, etc., but less studied in red spotted grouper [23,24,25,26,27,28]. Therefore, the present study aimed to explore the feasibility of using wheat gluten as a protein source in the low-FM-based diet for red spotted groupers by replacing FM or SPC with a large amount of wheat gluten.

## 2. Materials and Methods

### 2.1. Ingredients and Diets

Low-temperature dried FM, SPC, and wheat gluten were used as the main protein sources. The proximate compositions and amino acid profiles of FM, SPC, and wheat gluten are shown in Table 1. GWT is an ingredient blend containing 77.5% wheat gluten, 20.5% wheat flour, and 2.0% taurine. Eight isonitrogenous (441–456 g kg^−1^) and isocaloric (21.5–22.0 MJ kg^−1^) diets were formulated, including a control diet (R0, 20% FM, and 21.4% SPC), six diets with 33.3, 66.7, and 100% FM or SPC being gradually replaced by GWT (RF1, RF2, RF3, RS1, RS2, and RS3), and one diet with 50% FM and 50% SPC replaced by GWT (RFS). Diets were produced at the Feed Technology Laboratory of the Feed Research Institute, Chinese Academy of Agricultural Sciences in Beijing. All dry ingredients were ground in a hammer mill through a 0.18 mm screen, mixed, preconditioned, and extruded in a twin-screw extruder (MY56X2A, Muyang, Yangzhou, China) with a 2.0 mm die plate. All extruded pellets were dried to 950 g kg^−1^ dry matter at ambient temperature. Fish oil and soy lecithin were coated into pellets with a vacuum coater (ZJB-100). Feed formulations and chemical compositions of experimental diets are shown in Table 2.

### 2.2. Fish Management

After a 2-week acclimation, a 56-day feeding trial was conducted in the Fish Laboratory of the Sino-European Aquatic Nutrition and Feed Resource Institute, Zhejiang Ocean University, with an indoor seawater recirculation system. Before the feeding trial, 1200 healthy juveniles with a similar size (initial weight 9.55 ± 0.03 g) were selected, batch-weighed and randomly assigned to 24 circular 1000-L tanks (the water volume was approximately 750 L), with 50 fish per tank. Each tank was manually fed four times per day (08:00 a.m., 11:30 a.m., 3:00 p.m., and 6:30 p.m.), with 30 min per meal to ensure apparent satiety. After each feeding, the remaining feed pellets in each tank were counted according to the method of Zhang et al. [29] and siphoned out immediately. The daily feeding rate was tentatively set at 10% in excess based on the average intake over the past three days, and fish could receive more feed if they showed signs of feeding. During the feeding, each tank was supplied with seawater at a flow rate of 8–9 L min^−1^ and additional aeration via a nano-air-stone to ensure the dissolved oxygen was above 6.0 mg L^−1^. One-third of the seawater in each tank was exchanged daily with an equal amount of new sand-filtered seawater through the filter system. Artificial photoperiod was adopted, average water temperament and salinity were 27.9 °C and 28 ppt throughout the whole trial, and the photoperiod was 12D:12L.

### 2.3. Sampling

Before the feeding trial, 3 × 12 fish of similar size were selected and stored at −20 °C for whole-body composition analysis. After a 28-day feeding trial, five fish per tank were weighed to calculate weight gain. At the end of the 56-day feeding trial, fish in the same tank were counted and batch-weighed after anaesthesia with MS-222 (90 mg L^−1^). Five fish per tank were randomly selected to determine body length and weight individually, and then the liver and carcass were weighed to calculate morphologic indexes. Five fish in each tank were taken as whole-body samples. Blood samples were collected from the caudal vein of fish with disposable and disinfected injectors, centrifuged at 4 °C for 10 min (4000× *g*), and then stored at −80 °C until analysis. Liver from six fish per tank were collected for analyses of antioxidant parameters.

### 2.4. Analysis

Initial and final whole-body samples from the same tank were cut into pieces and grounded with a meat grinder, then autoclaved (YXQ-LS, Xunbo, Shanghai, China) at 120 °C for 30 min, homogenised by a homogeniser (DS-1, Shanghai, China), and oven-dried (Jinghong, China) at 80 °C. Whole-body samples were finely ground with a pestle and mortar until all samples passed through the 0.9 mm screen. Dried whole fish and feed samples were analysed for dry matter (105 °C to constant weight), protein (Kjeldahl’s method), lipid (Soxhlet Extraction System, Jingke, Shanghai, China), amino acids (amino acid analyser, L-8900, Hitachi, Tokyo, Japan), ash (550 °C, overnight) and energy (Phillipson Microbomb Calorimeter, Gentry Instruments Inc., Aiken, SC, USA) based on the previous studies [17]. Contents of total protein (TP), triglycerides (TG), cholesterol (TC), glucose (GLU), and activities of glutamate–pyruvate transaminase (ALT), glutamate–oxaloacetate transaminase (AST) and superoxide dismutase (SOD) were determined by the BECKMAN COULTER AU5800 automatic biochemical analyser. In addition, superoxide dismutase (SOD) activity and MDA (malonaldehyde) content in the liver were determined using commercial kits (Nanjing Jiancheng Bioengineering Institute, Nanjing, China) according to the manufacturer’s instructions.

### 2.5. Calculation and Statistical Analysis

The parameters were calculated as follows:

Weight gain (WG, g) = final body weight − initial body weight;

Feed conversion ratio (FCR, g DM ingested (g gain)^−1^) = feed intake/(final mean body weight—initial mean body weight);

Hepatosomatic index (HSI, %) = liver weight/body weight × 100;

Viscerosomatic index (VSI, %) = viscera weight/body weight × 100;

Condition factor (CF, g/cm^3^) = body weight/body length^3^ × 100;

Protein retention efficiency (PRE, %) = protein gain/protein intake × 100;

Energy storage ratio (ERE, %) = energy gain/energy intake × 100.

All data are presented as means ± S.E.M. (n = 3) and assessed by one-way analysis of variance (ANOVA) using the Origin 8.0 Pro SR4 (Origin Lab. Co., Northampton, MA, USA) software. Results were considered to be significant at *p* < 0.05. Moreover, a follow-up trend analysis using orthogonal polynomial contrasts was performed to determine whether the significances were linear and/or quadratic. Limitations of this study are the post hoc analysis performed after ANOVA and the lower regression index R in several analyses.

## 3. Results

### 3.1. Growth Performance, Feed Utilization and Morphologic Indexes

No fish died during the 56-day feeding trial. Furthermore, fish in all groups showed high growth performance and feed utilisation (Table 3). On the 56-day feeding trial, significant differences were recorded in FI, WG and FCR among different groups. Fish fed with the RF3 diet had the highest WG (~240%), whereas fish fed with the diet without wheat gluten supplementation (R0 diet) showed the lowest WG (~150%) among all treatments. Fish fed with RF3, RS2 and RFS diets showed a higher value of FI (24–25 g per fish^−1^) than those fed the R0 diet (~19 g per fish^−1^) (*p* < 0.05). The lowest value of FCR was observed in the RF3 and RS2 groups, while the highest value was found in the R0 group. However, no significant differences were recorded in FI, WG, and FCR among all groups on the 28-day feeding trial (*p* > 0.05). Similarly, no significant differences were found in HSI, VSI, and CF among fish fed different experimental diets (Table 4).

**Figure 1:**
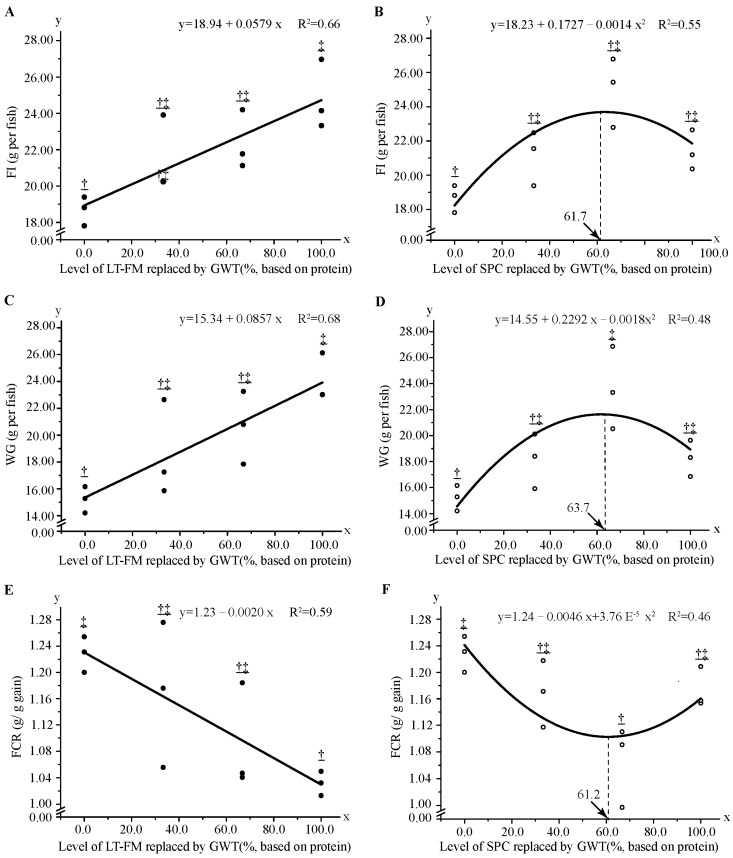
Regression analysis of growth performance and feed utilisation in red spotted grouper fed diet with different GWT inclusion levels. Data were analysed by one-way ANOVA followed by Tukey multiple comparisons, with different underlined symbols indicating significant differences (*p* < 0.05) among treatments. (**A**,**C**,**E**) show the regression analysis of replacing LT-FM with GWT on growth performance and feed utilisation. (**B**,**D**,**F**) represent the regression analysis of replacing SPC with GWT on growth performance and feed utilisation. GWT, a mixture of wheat gluten, wheat, and taurine; LT-FM, low-temperature dried fish meal; SPC, soy protein concentrate.

To further investigate the effects of wheat gluten replacing FM and SPC in red spotted grouper, the regression method was used to analyse the correlation between wheat gluten supplementation level and WG, FI, and FCR. The results showed that the above indices varied linearly with decreasing dietary FM level but quadratic polynomially with reducing dietary SCP level (Figure 1). Specifically, based on the first-order fitting equation, the level of dietary GWT and SPC were estimated to be 207 g kg^−1^ and 214 g kg^−1^, respectively (Figure 1A,C,E). The optimum levels of wheat gluten according to FI, WG, and FCR were 128 g kg^−1^ (GWT inclusion level was ~61.7%), 131 g kg^−1^ (~63.7%) and 127 g kg^−1^ (~61.2%) based on the second-order regression equation with 200 g kg^−1^ FM in the feed formula, respectively (Figure 1B,D,F).

### 3.2. Body Compositions and Nutrient Retentions

**Figure 2:**
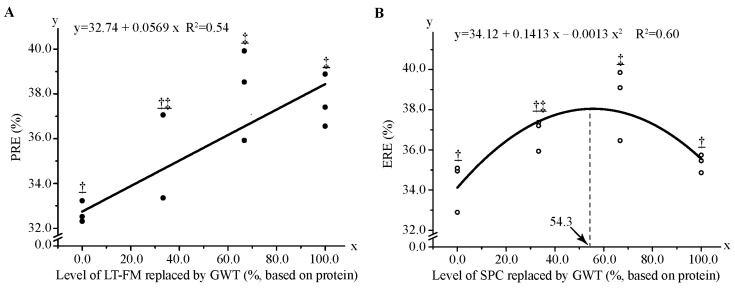
Regression analysis of protein retention efficiency (**A**) and energy retention efficiency (**B**) in red spotted grouper fed diet with different GWT inclusion levels. Data were analysed by one-way ANOVA followed by Tukey multiple comparisons, with different underlined symbols indicating significant differences (*p* < 0.05) among treatments. ERE, energy retention efficiency; GWT, a mixture of wheat gluten, wheat and taurine; LT-FM, low-temperature dried fish meal; PER, protein retention efficiency; SPC, soy protein concentrate.

The proximate compositions in whole-body and nutrient retentions are shown in Table 5. Contents of protein, fat and ash, and energy level and ERE were not significantly different among all treatments (*p* > 0.05). Fish fed with the diets RF2 and RF3 had the highest PRE, whereas the R0 diet showed the lowest value. PRE increased linearly with the increase of dietary FM substitution level (Figure 2A), while ERE increased quadratically with increasing dietary SPC replacement level (Figure 2B). The maximum ERE was obtained when 54.3% SPC was replaced by wheat gluten based on the second-order regression equation.

### 3.3. Serum and Liver Physiological and Antioxidant Parameters

**Figure 3:**
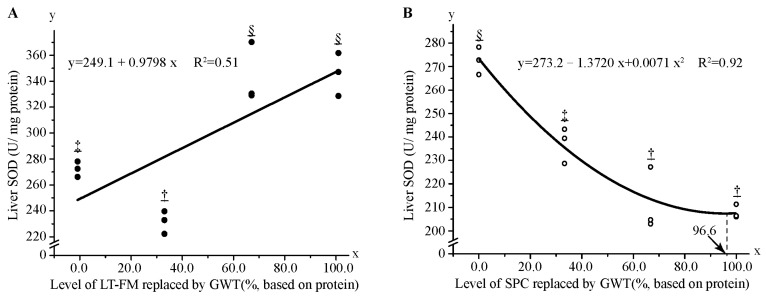
Regression analysis of liver superoxide dismutase (SOD) activity in red spotted grouper fed diet with different GWT inclusion levels. Data were analysed by one-way ANOVA followed by Tukey multiple comparisons, with different underlined symbols indicating significant differences (*p* < 0.05) among treatments. (**A**,**B**) show the regression analysis of replacing LT-FM and SPC with GWT on liver SOD activity, respectively. GWT, a mixture of wheat gluten, wheat and taurine; LT-FM, low-temperature dried fish meal; SPC, soy protein concentrate.

After the 56-day feeding trial, no significant differences were found in serum TP, TC, TG, GLU, ALT, AST, SOD, and MDA among fish fed diets with FM and/or SPC gradually replaced by GWT (Table 6). However, according to a linear regression model (y = 249.1 + 0.9798x, R^2^ = 0.51), liver SOD activity markedly increased among fish fed diets, with FM gradually replaced by wheat gluten. When SPC was gradually replaced by wheat gluten, liver SOD activity decreased in a quadratic polynomial model (y = 273.2 − 1.372x + 0.0071x^2^, R^2^ = 0.92) (Figure 3).

## 4. Discussion

The present study clearly showed the feasibility of wheat gluten as the main protein source in low-FM diets (20%) for red spotted grouper. Fish fed a diet with GWT supplementation showed a WG value of 190–250%, while the WG in fish fed diet R0 was around 159%. This growth rate was slightly lower than the study by Wang et al. [18], who selected similar size fish (~7.88 g) and a fish-meal-casein-based diet. Furthermore, the effect of diet on the growth of aquatic animals was time-accumulating. The growth performance of fish was not significantly different on the 28th feeding day, but it was markedly different on the 56th feeding day. At the end of the feeding, an increase of WG was observed in fish fed GWT supplementation diets compared with the R0 diet, including that wheat gluten could improve the growth of red spotted grouper juveniles. Similar results were also reported in a previous study, in which wheat gluten partly replaced dietary FM in large yellow croaker [16]. However, different results were reported for European sea bass [15] and Atlantic halibut [14], with their growth not significantly affected by dietary wheat gluten levels. Increased growth due to the supplementation of GWT could be attributed to the higher feed intake and utilisation. Some supplements were used in this study, such as krill meal, taurine and lecithin, which can ensure the attractivity of feed. Krill meal is often used as a feed stimulant due to its high palatability to marine fish, especially the high plant protein content for carnivorous fish. Plant proteins are deficient in taurine, which is necessary for groupers, especially in the high plant protein diet [30]. Previous studies have demonstrated that taurine is a critical nutrient in plant-based diets for Japanese flounder [31], Cobia Rachycentron canadum [32], white seabass *Atractoscion nobilis* [33], sablefish *Anoplopoma fimbria* [34] and meagre *Argyrosomus regius* [35]. The incorporation of lecithin in diets with low or no FM had positive effects on the growth performance of rainbow trout [36] and tinfoil barb *Barbonymus schwanenfeldii* [37]. Secondly, wheat gluten is not only rich in protein but has a much higher digestibility of protein and amino acids than fishmeal and soy protein concentration [11]. Moreover, high levels of anti-nutritional factors due to the increased plant protein level in the diet caused a decrease in feed nutritive value, feed palatable and feed utilisation. However, wheat gluten has fewer antinutritional factors, especially anti-palatability factors, compared with other plant protein sources, such as saponins, alkaloids, fibre, and phytic acid [38]. Thus, all these studies observed good feed intake and high feed utilisation when replacing FM and/or SPC with wheat gluten. Based on the nutritional properties of wheat gluten, balancing the dietary amino acid profile and adding appropriate starch levels may help groupers to adapt to high levels of wheat gluten.

Plant protein sources usually lack one or more essential amino acids. Compared to the FM, wheat gluten is limited in lysine, methionine, arginine, and threonine (Table 1) [39]. Lysine is the most limited amino acid in wheat gluten. The level of lysine in this experiment is approximately 26.0 g kg^−1^ diet (57.7 g kg^−1^ dietary protein), which is higher than the requirement of hybrid grouper (21.6 g kg^−1^ diet, corresponding to 40.5 g kg^−1^ dietary protein) [40], but lower than the *E. coioides* (28.3 g kg^−1^ diet, corresponding to 55.6% of dietary protein) [41]. Methionine is the second most limited amino acid, and its level in this experimental is 27.6–30.3 g kg^−1^ diet (61.3–67.3 g kg^−1^ dietary protein), higher than the optimum requirement of *E. coioides* (13.1 g kg^−1^ diet, corresponding to 27.3 g kg^−1^ dietary protein) [42]. Supplementation of limited amino acids in wheat gluten-supplemented diets is beneficial in improving feed utilisation, which is consistent with previous studies in which crystal amino acids were supplemented in plant protein-based diets [1,43,44]. Moreover, the optimal level of wheat gluten substitution for SPC was estimated to be 61% in this experiment (Figure 1B,D,F). Starch is the main component of wheat flour. Generally, starch is poorly utilised by aquatic animals, and its digestibility decreases with increasing dietary starch levels in almost fish and shrimp species. According to the results of a previous study, the tolerable level of wheat flour in red spotted grouper was 286 g kg^−1^ diet, which is close to 280 g kg^−1^ diet reported in hybrid grouper (*E. lanceolatus* 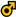 × *E. fuscoguttatus* 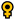) [45], but higher than 72.2 g kg^−1^ diet (incorporation with a cellulose content of ~250 g kg^−1^ diet) and 246 g kg^−1^ diet reported in *E. akaara* [19] and *E. malabaricus* [46] respectively. In this study, higher dietary starch decreased growth performance and feed utilisation in red sported grouper, which was consistent with previous studies on Nile tilapia *Oreochromis niloticus* [47], gibel carp *Carassius auratus* var. *gibelio* [48], grass carp *Ctenopharyngodon idella* [49], hybrid grouper *E. fuscoguttatus* 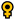 × *E. lanceolatus* 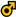 [50] and largemouth bass *Micropterus salmoides* [51].

Proximate composition and nutrition retention among fish fed diets with or without wheat gluten supplementation are shown in Table 4. The crude protein was about 180 g kg^−1^, similar to the value reported by Wang et al. [19] in *E. akaara*. Lipid content (~110 g kg^−1^) in whole-body was slightly higher than the level recorded by Wang et al. [19], mainly due to the higher dietary lipid level. However, no significant differences were observed in fish body compositions (moisture, crude protein, lipid, and ash) in this study, which is consistent with a study in red spotted grouper [18]. However, protein retention was significantly affected by different experimental diets. An increase in protein accumulation was observed in fish fed the diet with FM replaced by wheat gluten. Interestingly, the ERE was not evidently affected in the FM-replaced groups, while in the SPC-replaced groups, there was a secondary increase with increasing dietary wheat gluten levels. Fish, especially carnivorous fish, have limited availability of starch as an energy source. However, moderate wheat gluten could be used as an energy source has been demonstrated in Atlantic salmon *Salmo salar* [39], Atlantic cod *Gadus morhua* [52], and gilthead sea *bream Sparus aurata* [52].

In this study, no significant changes in serum indices were observed when FM or SPC was gradually replaced by wheat gluten. Similar to the results in European sea bass, no significant changes were observed in serum cholesterol when fed a wheat gluten-based diet compared with the FM diet [53]. In this study, liver SOD activity linearly increased with gradually increasing FM replacement levels (0, 1/3, 2/3, and 100%). However, no significant differences were observed in liver MDA content, indicating that wheat gluten has no deleterious effects on oxidative stress products in red spotted grouper. In contrast, a decrease in SOD activity and MDA content was observed in the group in which SPC was replaced by wheat gluten (Figure 3). Glutamate is a non-essential amino acid which is considered to be beneficial for the growth and antioxidant status of aquatic animals. Thus, increased SOD activity may be attributed to the high content of glutamate in wheat gluten, which is higher than FM and SPC [11].

## 5. Conclusions

The highest growth performance was observed in red spotted grouper fed diets with 100% fishmeal or 66.7% soy protein concentration replaced by wheat gluten. Regression analysis of weight gain and feed conversion efficiency suggested that the optimal levels of wheat gluten substitution for fish meal or soy protein concentration were 20.7% and 13.1%, respectively. The results of this study provide new insights for the development of a fishmeal-free formulation for red spotted grouper.

## Figures and Tables

**Table 1:** Compositions and amino acid profiles of wheat gluten, soy protein concentration, and fish meal in the experiment (DM).

Ingredients	Fish Meal ^†^	Wheat Gluten ^‡^	Soy Protein Concentration ^§^
Compositions, kg^−1^			
Dry matter, g	913	935	925
Crude protein, g	748	837	694
Crude fat, g	117	44	47
Starch, g	-	74	-
Ash, g	122	10	58
Gross energy, MJ	21.8	22.0	20.1
Essential amino acids (EAA), g (16 g N)^−1^			
Arg	5.5	3.6	7.4
His	1.7	2.0	2.5
Leu	7.0	7.0	8.1
Ile	3.8	3.5	4.6
Lys	7.3	1.8	6.7
Met	2.6	1.4	0.9
Phe	3.8	5.2	5.3
Thr	4.1	2.5	4.1
Tyr	3.2	3.3	3.5
Val	4.5	3.8	4.7
Total EAA ^¶^	43.3	34.1	47.9
Non-essential amino acids (NEAA), g (16 g N)^−1^			
Ala	6.1	2.7	4.4
Asp	8.4	3.2	11.6
Cys	0.8	1.8	0.3
Glu	13.1	35.2	19.1
Gly	5.8	3.4	4.2
Pro	4.2	12.8	5.0
Ser	4.0	4.6	5.2
Total NEAA	42.3	63.8	49.8
Total AA ^¶^	85.6	97.9	97.6

^†^ TripleNine^®^, low-temperature dried fish meal, Esbjerg, Denmark. ^‡^ AMYGLUTEN 110, Syral Belgium N.V., Aalst, Belgium. ^§^ YIHAI^®^, Wilpromil, Goldensea Grain and Oil Industry Co., Ltd., Wilmar, Qinhuangdao, China. ^¶^ Trp excluded.

**Table 2:** Feed formulations and chemical compositions of experimental diets (DM).

Ingredients, g kg^−1^	R0	RF1	RF2	RF3	RS1	RS2	RS3	RFS
Constant feed ingredients ^†^	221.5	221.5	221.5	221.5	221.5	221.5	221.5	221.5
GWT ^‡^	-	69.0	138.0	207.0	69.0	138.0	207.0	207.0
Fish meal ^§^	200.0	133.0	66.0	-	200.0	200.0	200.0	100.0
Soy protein concentrate	214.0	214.0	214.0	214.0	142.0	71.0	-	107.0
Wheat flour	266.4	250.4	234.6	217.8	262.9	259.1	255.4	236.3
Fish oil	84.0	89.0	94.0	99.0	84.0	84.0	84.0	92.0
Mono calcium Phosphate ^¶^	14.0	17.0	20.0	23.0	14.5	14.5	14.5	19.0
L-Lysine ^††^	-	3.7	7.4	11.0	3.1	6.1	9.1	10.0
DL-Methionine ^††^	-	0.4	0.8	1.2	-	-	-	0.2
L-Arginine ^††^	-	1.1	2.1	3.1	2.1	4.2	6.2	4.7
L-Threonine ^††^	-	0.8	1.5	2.3	0.8	1.5	2.2	2.2
Analysed content, kg^−1^								
Dry matter, g	951	954	957	956	957	957	957	955
Crude protein, g	441	447	444	449	447	451	456	454
Crude fat, g	135	136	139	142	140	141	131	140
Ash, g	76	69	61	54	72	68	54	59
Gross energy, MJ	21.5	21.6	21.6	22.0	21.5	21.7	21.8	22.0
Essential amino acid, g (16 N)^−1^								
Arg	6.5	6.5	6.6	6.4	6.5	6.3	6.8	6.2
Ile	4.1	3.9	3.9	3.8	3.9	3.7.	3.7	3.5
Leu	7.2	7.0	7.2	7.0	7.0	6.7	6.9	6.6
Lys	6.1	6.0	6.1	6.1	6.1	5.9	6.2	5.9
Met + Cys	2.1	2.0	2.1	2.1	2.0	2.0	2.5	1.9
Phe + Tyr	7.4	7.3	7.7	7.7	7.1	6.7	7.3	6.8
Thr	3.8	3.8	3.8	3.7	3.8	3.7	3.8	3.5
Val	4.4	4.2	4.1	4.0	4.2	4.0	4.1	3.8
Total essential amino acid	41.7	40.7	41.5	40.7	40.6	39.0	41.4	38.2
Non-essential amino acid, g (16 N)^−1^								
Ala	4.7	4.3	4.0	3.5	4.6	4.2	4.3	3.7
Asp	9.9	8.8	8.6	7.8	8.8	7.8	7.1	6.9
Glu	16.3	18.7	21.0	22.9	18.0	19.1	22.1	21.4
Gly	5.0	4.5	4.3	3.9	4.7	4.5	4.69	4.0
Pro	4.7	5.4	6.1	7.0	5.5	6.0	7.2	7.2
Ser	4.4	4.4	4.6	4.5	4.3	4.2	4.3	4.2
Total non-essential amino acid	45.0	46.0	48.6	49.6	46.0	45.7	49.7	47.3
Total amino acid	86.6	86.8	90.1	90.4	86.5	86.6	90.9	85.6

^†^ Constant feed ingredients, kg^−1^: Soybean meal 70 g (FENGYUAN^®^, Goldensea Grain and Oil Industry Co., Ltd., Wilmar, Qinhuangdao, China); Peanut meal 70 g (FENGYUAN^®^, Goldensea Grain and Oil Industry Co., Ltd., Wilmar, Qinhuangdao, China); Krill meal 50 g (QRILLTM, Antarctic Krill Meal, Aker BioMarine, Oslo, Norway); Soy lecithin 20 g (FENGYUAN^®^, Goldensea Grain and Oil Industry Co., Ltd., Wilmar, Qinhuangdao, China); Premix 10 g (Vitamin premix (mg kg^−1^ diet): vitamin A, 20; vitamin B_1_, 12; vitamin B_2_, 10; vitamin B_6_, 15; vitamin B_12_, 8; niacinamide, 100; ascorbic acid, 1000; calcium pantothenate, 40; biotin, 2; folic acid, 10; vitamin E, 400; vitamin K_3_, 20; vitamin D_3_, 10; inositol, 200; corn protein powder, 150; Mineral premix (mg kg^−1^ diet): CuSO_4_ · 5H_2_O, 10; FeSO_4_ · H_2_O, 300; ZnSO_4_ · H_2_O, 200; MnSO_4_ · H_2_O, 100; KI (10%), 80; Na_2_SeO_3_ (10% Se), 67; CoCl_2_ · 6H_2_O (10% Co), 5; NaCl, 100; zeolite, 638; Vitamin premix: mineral premix = 2:1); Choline Cl 1.5 g (Be-long corporation, Nanjing, China). ^‡^ Mixture of vital wheat gluten, wheat flour, and taurine (ratio: 77.5%, 20.5%, and 2.0%). Wheat flour, BLUEKEY^®^, Beijing Grain and Oil Industry Co., Ltd., Wilmar, Beijing, China. Taurine-JP8, Qianjiang Yongan Pharmaceutical Co., Ltd., Qianjiang, China. ^§^ Aidayufen Co., Ltd., Rongcheng, China. ^¶^ MCP22, mono calcium phosphate, feed grade, Suntran Industrial Group Ltd., Hefei, China. ^††^ Siwei Development Group Ltd., Hangzhou, China.

**Table 3:** Growth performance and feed utilisation of red spotted grouper fed diet with different GWT inclusion levels.

Diets	Feed Intake, g DM Fish^−1^		Weight Gain, g Fish^−1^		Feed Conversion Ratio, g DM Ingested (g Gain)^−1^	
	0–28 Days	0–56 Days	0–28 Days	0–56 Days	0–28 Days	0–56 Days
R0	11.9 ± 0.28	18.7 ± 0.46 ^†^	12.2 ± 0.36	15.2 ± 0.57 ^†^	0.98 ± 0.01	1.23 ± 0.02 ^‡^
RF1	12.4 ± 0.16	21.5 ± 1.22 ^†‡^	12.5 ± 0.24	18.6 ± 2.07 ^†‡^	0.99 ± 0.01	1.17 ± 0.06 ^†‡^
RF2	12.0 ± 0.04	22.4 ± 0.94 ^†‡^	12.9 ± 0.14	20.6 ± 1.56 ^†‡^	0.93 ± 0.01	1.09 ± 0.05 ^†‡^
RF3	12.1 ± 0.25	24.8 ± 1.10 ^‡^	12.5 ± 0.35	24.1 ± 1.04 ^‡^	0.96 ± 0.02	1.03 ± 0.01 ^†^
RS1	12.3 ± 0.52	21.1 ± 0.92 ^†^	12.1 ± 0.73	18.2 ± 1.22 ^†^	1.02 ± 0.03	1.17 ± 0.03 ^†‡^
RS2	13.1 ± 0.17	25.0 ± 1.17 ^‡^	14.2 ± 0.33	23.6 ± 1.83 ^‡^	0.92 ± 0.01	1.07 ± 0.03 ^†‡^
RS3	12.4 ± 0.61	21.4 ± 0.67 ^†‡^	12.7 ± 1.01	18.3 ± 0.80 ^†‡^	0.98 ± 0.03	1.17 ± 0.02 ^†‡^
RFS	12.7 ± 0.20	23.9 ± 1.59 ^‡^	13.4 ± 0.29	22.0 ± 2.28 ^†‡^	0.95 ± 0.02	1.10 ± 0.05 ^†‡^
ANOVA *p*	0.27	0.01	0.15	0.01	0.05	0.03

Data were represented as means ± S.E.M. Different superscript symbols indicate significant differences (*p* < 0.05) among treatments.

**Table 4:** Morphologic indexes of red spotted grouper fed diet with different GWT inclusion levels.

Parameters	R0	RF1	RF2	RF3	RS1	RS2	RS3	RFS	*p*
HSI, %	2.08 ± 0.25	1.76 ± 0.25	2.16 ± 0.16	2.03 ± 0.20	1.76 ± 0.22	1.97 ± 0.20	2.08 ± 0.15	1.81 ± 0.26	0.61
VSI, %	13.8 ± 0.23	13.8 ± 0.28	13.4 ± 0.46	14.3 ± 0.35	13.5 ± 0.48	13.4 ± 0.30	13.2 ± 0.19	13.2 ± 0.39	0.31
CF, g/cm^3^	2.72 ± 0.04	2.84 ± 0.08	2.89 ± 0.07	2.93 ± 0.06	2.80 ± 0.06	2.80 ± 0.04	2.96 ± 0.09	2.81 ± 0.10	0.27

Data were represented as means ± S.E.M.

**Table 5:** Body compositions and nutrition retentions of red spotted grouper fed diet with different GWT inclusion levels.

Diets	Dry Matter, g kg^−1^	Proximate Composition, g kg^−1^			Energy, KJ kg^−1^	Retention, %	
		Protein	Lipid	Ash		Protein (PRE)	Energy (ERE)
R0	331 ± 5.78	176 ± 1.53	106 ± 2.85	49.7 ± 2.24	8.06 ± 0.12	32.7 ± 0.28 ^†^	34.3 ± 0.71
RF1	326 ± 5.29	174 ± 5.24	103 ± 3.84	47.9 ± 2.37	8.43 ± 0.34	33.9 ± 1.66 ^†‡^	34.7 ± 2.33
RF2	333 ± 9.45	178 ± 2.73	111 ± 4.37	44.3 ± 2.78	8.26 ± 0.20	38.1 ± 1.17 ^‡^	38.6 ± 1.18
RF3	324 ± 5.78	173 ± 2.40	110 ± 2.19	40.5 ± 1.50	8.24 ± 0.14	37.6 ± 0.68 ^‡^	39.4 ± 1.19
RS1	335 ± 0.33	179 ± 1.67	110 ± 1.86	46.7 ± 0.35	8.30 ± 0.06	35.9 ± 1.20 ^†‡^	36.8 ± 0.45
RS2	327 ± 3.18	175 ± 0.88	108 ± 2.73	44.5 ± 1.37	8.19 ± 0.13	36.4 ± 1.04 ^†‡^	38.5 ± 1.03
RS3	333 ± 1.00	182 ± 0.88	105 ± 1.00	46.1 ± 1.13	8.17 ± 0.02	35.7 ± 0.74 ^†‡^	35.4 ± 0.26
RFS	330 ± 4.73	178 ± 4.33	109 ± 4.70	43.5 ± 2.75	8.22 ± 0.11	36.5 ± 0.64 ^†‡^	37.3 ± 1.69
ANOVA *p*	0.79	0.53	0.64	0.11	0.88	0.03	0.08

Data were represented as means ± S.E.M. Different superscript symbols indicate significant differences (*p* < 0.05) among treatments.

**Table 6:** Biochemical indices in plasma and liver of red spotted grouper fed diet with different GWT inclusion levels.

Parameters	Diets								*p*
	R0	RF1	RF2	RF3	RS1	RS2	RS3	RFS	
Plasma									
TP, g/L	32.5 ± 1.36	28.7 ± 5.14	34.3 ± 2.17	30.7 ± 1.03	34.4 ± 1.60	33.2 ± 0.76	34.2 ± 0.27	32.7 ± 1.89	0.61
TG, mmol/L	4.20 ± 1.60	2.73 ± 1.10	2.43 ± 0.42	1.76 ± 0.34	5.04 ± 2.03	4.20 ± 3.03	4.01 ± 0.22	1.88 ± 0.88	0.70
CHOL, mmol/L	3.94 ± 0.72	2.61 ± 0.69	2.41 ± 0.26	1.69 ± 0.02	4.00 ± 0.52	2.72 ± 0.75	3.14 ± 0.11	2.60 ± 0.31	0.07
GLU, mmol/L	7.96 ± 0.32	6.42 ± 0.92	6.01 ± 0.85	5.51 ± 0.56	7.52 ± 1.92	6.22 ± 1.68	6.31 ± 0.48	6.39 ± 1.09	0.81
ALT, U/L	1.83 ± 0.88	7.50 ± 4.54	2.17 ± 0.17	3.17 ± 0.33	2.00 ± 0.29	3.17 ± 0.60	2.67 ± 0.17	2.67 ± 0.93	0.37
AST, U/L	122 ± 11.1	102 ± 32.5	142 ± 14.1	128 ± 15.4	117 ± 35.0	119 ± 15.2	132 ± 10.8	94.5 ± 22.3	0.80
SOD, U/mL	21.0 ± 0.42	22.4 ± 0.19	22.0 ± 0.44	22.3 ± 0.66	20.5 ± 0.19	21.7 ± 0.39	20.8 ± 0.22	21.5 ± 0.16	0.12
Liver									
SOD, U/mg prot	273 ± 3.38 ^‡^	233 ± 4.95 ^†^	342 ± 13.2 ^§^	345 ± 9.40 ^§^	237 ± 4.36 ^†^	212 ± 7.80 ^†^	208 ± 1.70 ^†^	283 ± 3.65 ^‡^	<0.01
MDA, nmol/mg prot	1.74 ± 0.04	1.52 ± 0.02	2.17 ± 0.01	2.08 ± 0.38	2.77 ± 0.66	1.58 ± 0.01	1.54 ± 0.01	1.40 ± 0.03	0.05

Data were represented as means ± S.E.M. Different superscript symbols indicate significant differences (*p* < 0.05) among treatments.

## Data Availability

Data are available on request due to privacy. The data presented in this study are available on request from the corresponding author.
